# The lncRNA Rhno1/miR-6979-5p/BMP2 Axis Modulates Osteoblast Differentiation

**DOI:** 10.7150/ijbs.38930

**Published:** 2020-03-12

**Authors:** Yuan Xiong, Lang Chen, Chenchen Yan, Yori Endo, Bobin Mi, Guohui Liu

**Affiliations:** 1 Department of Orthopaedics, Union Hospital, Tongji Medical College, Huazhong University of Science and Technology, Wuhan 430022, China.; 2 Division of Plastic Surgery, Brigham and Women's Hospital, Harvard Medical School, Boston, 02215, USA.

**Keywords:** LncRNA, miRNA, mRNA, Fracture, BMP2

## Abstract

The roles of long non-coding RNAs (lncRNAs) and micro RNAs (miRNAs) as regulators of mRNA expression in various diseases have recently been reported. Osteoblast differentiation is the vital process which mediates bone formation and fracture healing. In present study, we found microRNA-6979-5p (miR-6979-5p) to be the most differentially expressed miRNA between normal bone and calluses of mice, and overexpression of miR-6979-5p was negatively associated with osteoblast differentiation. Through luciferase assays, we found evidence that bone morphogenetic protein 2 (BMP2) is a miR-6979-5p target gene that positively regulates osteoblast differentiation. We further identified the lncRNA Rhno1 as a competing endogenous RNA (ceRNA) of miR-6979-5p, and we verified that it was able to influence osteoblast differentiation both *in vitro* and *in vivo*. In summary, our data indicates that the lncRNA Rhno1/miR-6979-5p/BMP2 axis is a significant regulatory mechanism controlling osteoblast differentiation, and it may thus offer a novel therapeutic strategy for fracture healing.

## Introduction

Fracture healing is a complex physiological process that occurs in individuals after injury, and osteoblast differentiation is a vital component of this process 1,2. Previous studies have suggested that osteogenic differentiation is triggered via the up- or down-regulation of hormones and growth factors that ultimately modulate osteoblast differentiation and proliferation 3-5. For example, orexin signals through orexin receptor 2 in the brain to enhance bone formation, and exerts opposing regulatory roles in the context of osteoporosis 6. Similarly, targeting integrins had been reported to be a potential therapeutic strategy for counteracting bone loss 7. In recent years, there has been increasing research regarding the field of osteoblast differentiation, and specifically of the regulatory roles of the lncRNA/miRNA/mRNA axis in this process 8-10.

LncRNAs are long RNAs that lack coding ability, but which are transcribed and processed 11,12. There is now clear evidence that many lncRNAs play a critical role in regulation of gene expression via transcriptional or post-transcriptional mechanisms, and thereby participate in physiological and pathological processes 13,14. lncRNAs have also been reported to function as competing endogenous RNAs (ceRNAs), binding to specific miRNA response elements to effectively sequester specific miRNAs, thereby regulating processes such as osteogenic-related gene expression 15. This strategy effectively prevents miRNAs from binding to their target mRNA molecules, which they are normally able to repress the translation of in a highly specific manner via binding to their 3'-untranslated region (3'-UTR) 16. This lncRNA/miRNA/mRNA axis had been reported to widely exist in various disease contexts, but there is limited research regarding how this axis functions in the context of osteogenic differentiation, making the investigation of these functions and the underlying molecular mechanisms an important research pursuit.

Rhno1 (aka RHINO, RAD9-Hus1-Rad1 Interacting Nuclear Orphan) was reported as a factor to DNA damage checkpoint signaling, which physically couples the 9-1-1 (RAD9-HUS1-RAD1) checkpoint clamp to DNA Topoisomerase II Binding Protein 1 17. Ensemble mirror database (http://useast.ensembl.org) was used to search for the details of Rhno1. The results showed that Rhno1 have a lot of transcript ID and many of them have the ability of protein coding. However, a non-coding transcript ID (ENSMUST00000132604.1) named Rhno1-204 can also be found in this database. Thus, in the present research, we used this transcript ID as lncRNA Rhno1 and explore the association between lncRNA Rhno1 and osteoblast differentiation. From literature reviewing, few studies concerning miR-6979-5p, we then search for the miRBase database (www.mirbase.org) to get the more details of miR-6979-5p and the results indicated that the accession number of miR-6979-5p is MI0022827, and the sequence is: GGGGAGGCGCAGAGACUGAGCUGCUCAGUGCGCGCUUGUGUCUGUCUGGCUCCCAG.

BMP2, as a significant member in the transforming growth factor-β family, has demonstrated a regulatory role in the formation of cartilage 18. Furthermore, previous research reported that BMP2 can accelerate fracture healing by stimulating the bone marrow mesenchymal stem cells differentiate into osteoblasts 19. Additionally, it is shown that BMP2 can induce the osteoclast activity and the remodeling process of fracture healing 20. And a recent study reported that the expression of BMP2 was significantly decreased in patients with bone none union compared with fracture healing patients 21. Therefore, BMP2 is a key target gene for fracture healing and the formation of callus.

We aimed to investigate the ability of the lncRNA Rhno1/miR-6979-5p/BMP2 axis to regulate osteoblast differentiation *in vivo* and *in vitro*. We found that miR-6979-5p exerts a negative effect on osteoblast differentiation via directly targeting BMP2, and local injection of miR-6979-5p into fracture sites was able to counteract fracture healing. Furthermore, lncRNA Rhno1 was able to promote osteoblast differentiation in MC3T3-E1 cells via serving as a miR-6979-5p ceRNA, and local injection of lncRNA Rhno1 was able to enhance fracture healing *in vivo*. This is the first report to our knowledge to demonstrate the role of the lncRNA Rhno1/miR-6979-5p/BMP2 axis in osteoblast differentiation, and to outline the underlying mechanisms.

## Materials and Methods

### Animal fracture models

The Animal Research Committee of Union Hospital, Tongji Medical College, Huazhong University of Science and Technology approved all study experiments. Male C57BL/6J mice (age: 6 weeks) from the Center of Experimental Animal, Tongji Medical College, Huazhong University of Science and Technology were used for all experiments, with anesthesia conducted using 10% chloral hydrate (300mg/kg body weight). A longitudinal incision was made, and then blunt separation of the underlying muscles was performed to produce a murine femoral fracture model. Subsequently, we used a diamond disk to cut the femur in order to produce a transverse osteotomy in the mid-diaphysis region. All fractures were then stabilized using 23-gauge intramedullary needles. After 14 days, half of the mice were euthanized, and the calluses at the fracture location were harvested for subsequent analysis. Similarly, the calluses of remaining mice were harvested for subsequent analysis on day 21 post injury.

### Imaging of small animals *in vivo*

We performed small animal imaging at the Central laboratory of Union Hospital, Tongji Medical College, Huazhong University of Science and Technology (Wuhan Union Hospital; http://www.whuh.com/). On days 0, 4, 7, 10, and 14; post-local direct 100µl injections of 200 µM Cy3-labled agomiR-6979-5p were made (GenePharma, Shanghai, China). At 15 min post-injection, 200 µL of 1% chloral hydrate solution was used for anesthetization and were imaged using an In-Vivo FX PRO imaging system (BRUKER, Karlsruhe, Germany) with a 1-minute exposure time.

### Radiographic images

On days 7, 14, and 21 post-injury, all animals were given X-rays using an In-Vivo FX PRO imaging system (BRUKER, Karlsruhe, Germany) with a 10 second exposure time.

### micro-CT analysis

The fracture site was scanned using a BRUKER SkyScan 1176 scanner micro-CT system (BRUKER, Karlsruhe, Germany) to provide images at 2400 views, 5 frames/view, 37 kV, and 121mA, and three-dimensional images were rendered and evaluated using CT-Vox 2.1 (BRUKER Minimal Intensity Projection Software, Karlsruhe, Germany). Soft tissues were cleaned thoroughly. After scanning, calluses were preserved at -80°C for miRNA or protein extraction for qRT-PCR and western blotting, respectively. Parameters measured were measured by CT Analyser, Version: 1.15.4.0 evaluation software, as follows: bone volume (BV), total volume (TV), BV/TV, and bone mineral density (BMD).

### Cell cultures and transfection

MC3T3-E1 cells were grown in DMEM/F12 media (Hyclone, UT, USA, #SH30023.01B) with 10% FBS (Gibco, GI, USA, #10099141) and 1% penicillin/streptomycin (Hyclone, UT, USA, #SV30010). Transfection of agomiR-6979-5p, agomiR-NC, antagomiR-6979-5p, antagomiR-NC, lncRNA Rhno1, silncRNA Rhno1, and siRNA-NC (GenePharma, Shanghai, China) at 200uM was conducted using Lipofectamine 3000 (ThermoFisher Scientific, MA, USA, #L3000001) based on provided directions. Lipofectamine 3000 was also used to transfect cells with miRNAs or siRNA oligos. BMP2 siRNA, silncRNA Rhno1, and siRNA-NC (GenePharma, Shanghai, China) were transfected at 50 nM.

### Quantitative real-time PCR (qRT-PCR)

qRT-PCR was performed as in previous studies 42. Trizol (ThermoFisher Scientific, MA, USA, #L15596026) was used for total RNA extraction, after which a qPCR RT Master Mix (Toyobo, Osaka, Japan) was used for cDNA synthesis. The 2^-ΔΔCt^ method was used for relative gene expression quantification, with GAPDH and U6 used to normalize mRNA and miRNA expression. Primers are listed in Table 1.

### Western blotting

NETN buffer (20 mM TrisHCl, pH 8.0, 100 mM NaCl, 1 mM EDTA and 0.5% Nonidet P-40) was used to prepare cell lysates, which were separated via SDS-PAGE and transferred onto PVDF membranes. These were then blocked using 5% skim milk, and probed at 4°C overnight using primary antibodies, after which appropriate HRP-conjugated secondary antibodies (Aspen, Johannesburg, South Africa, #AS1058) were used for antigen detection. A chemiluminescence detection system (Canon, Tokyo, Japan, #LiDE110) was used to visualize protein based on provided directions. Antibodies used were as follows: anti-collagen I (1:500, Abcam, MA, USA, #ab34710), anti-ALP (1:1,000, Abcam, MA, USA, #ab95462), anti-Osteocalcin (1:500, Abcam, MA, USA, #ab93876), anti-RunX2 (1:500, Abcam, MA, USA, #ab23981), anti-BMP2 (1:2,000, CST, MA, USA), and anti-GAPDH (1:10,000, Abcam, MA, USA, #ab37168).

### Alkaline Phosphatase (ALP) Staining

A BCIP/NBT alkaline phosphatase color development kit (Beyotime, Shanghai, China, #C3206) used based on provided directions to assess ALP staining. Samples were washed twice in PBS, and then fixed with 10% formalin for 15 minutes. The BCIP/NBT liquid substrate was then applied to cells for 24 h. Samples were prepared in the dark at room temperature. Color change was obtained under a charge-coupled device (CCD) microscope, and a scanner (EPSON V600, Nagano prefecture, Japan) was used for imaging. Samples were analyzed in triplicate.

### Alizarin Red Staining

MC3T3-E1 cells were grown in osteogenic whole-media (Cyagen, Guangzhou, China, #HUXMA-90021) in 6-well plates to induce osteoblast mineralization. Cells were fixed with 10% formalin for 15 minutes, after which cells were washed using PBS and stained using 1 mL 0.5% alizarin red staining solution at room temperature for 15 min. Cells were then rinsed with distilled water for 5 minutes while shaking, and red mineralized nodules were assessed via CCD microscope and imaged (EPSON V600, Nagano prefecture, Japan). All experiments were repeated three times.

### Luciferase Reporter Assay

MC3T3-E1 cells were plated in 24-well plates and transfected using a dual-luciferase vector (BMP2 WT, BMP2 Mut, lncRNA Rhno1 WT and lncRNA Rhno1 Mut) as well as either miR-6979-5p mimics or negative control (NC mimics). Following a 24 h incubation, the dualluciferase reporter assay system (Promega, Wisconsin, USA) was utilized as a means of measuring luciferase activity.

### RNA immunoprecipitation (RIP) experiments

An RIP RNA-binding protein immunoprecipitation kit (Millipore, Billerica, MA, USA, #17-701) was used for RIP assays based on provided directions. Briefly, RIP lysis buffer (Solarbio, Beijing, China, #N8031) was used for MC3T3-E1 cell lysis, and lysates were combined with either anti-Argonaute2 (anti-Ago2) or anti-IgG control overnight at 4°C. Proteinase K was then used for sample treatment, and immunoprecipitated RNA was collected and analyzed via qRT-PCR.

### RNA-pull down assay

The Biotin RNA Labeling Mix (Roche, Basel, Switzerland, #11685597910) was used to labled isolated RNAs, while the T7 RNA polymerase (Ambion Life, TX, USA) was used for in vitro transcription. An RNeasy Plus Mini Kit (Qiagen, Munich, Germany, #74134) together with DNase I (Qiagen, Munich, Germany, #19101) was used for RNA purification. Cell lysates were then mixed together with these RNAs (positive control, negative control, or biotinylated), and magnetic beads were added to all samples followed by a room temperature incubation. Bead-bound RNA complexed were then eluted and extracted, after which RNA was analyzed via qRT-PCR.

### RNA fluorescent in situ hybridization

Cy3-labeled-lncRNA Rhno1 (GenePharma, Shanghai, China) and DAPI-labeled U6 probes (RiboBio, Guangzhou, China) were designed and synthesized. A FISH kit was used for RNA-FISH experiments based on provided protocols (ThermoFisher Scientific, MA, USA, #F32954).

### Histological analysis

Following decalcification, 7 µm thick paraffin-embedded tissue samples were prepared and subjected to hematoxylin-eosin (H&E) and Alcian blue staining. Sections were then imaged and measured with an Olympus BX51 microscope and a DP73 CCD Olympus Imaging System (Olympus Corporation, Tokyo).

### Statistical analysis

Data are means **±** SD, and GraphPad Prism 8.0 (GraphPad Software, Inc, La Jolla, CA) was used for all analyses unless otherwise noted. Three or more samples were compared via a one-way ANOVA with Tukey's post-hoc test, while two-tailed Student's tests were used to compare two groups. P < 0.05 was the threshold of significance.

## Results

### MiR-6979-5p inhibits fracture healing *in vivo*

**Figure 1:**
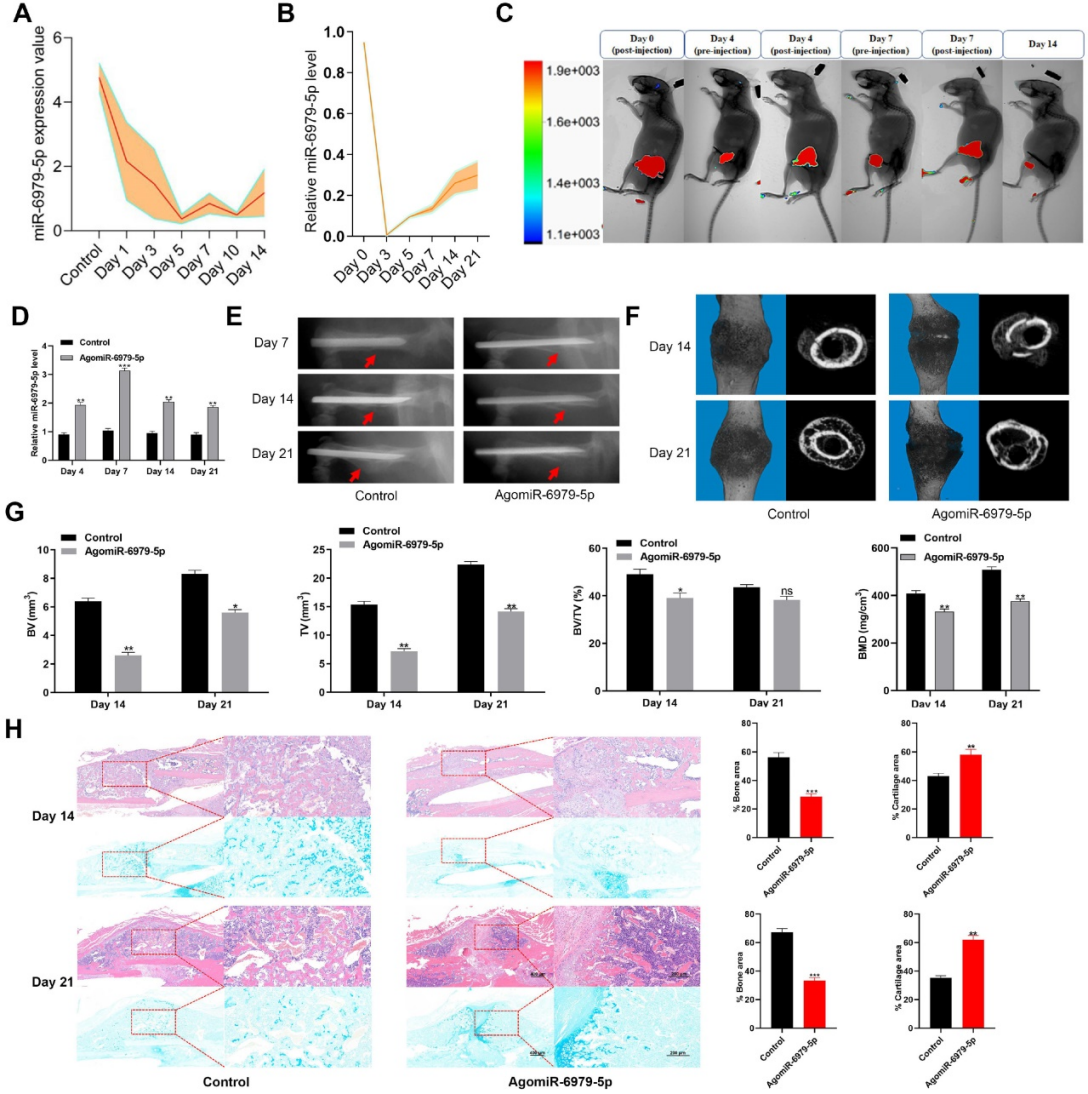
** Local administration of agomiR-6979-5p impaired healing in mice.** Levels of miR-6979-5p were generally decreasing in gene chips **(A)** and fracture animal models **(B)** at the early stages of fracture healing. **(C)** *In vivo* imaging of agomiR-6979-5p metabolism at the fracture sites. **(D)** High level of miR-6979-5p were found in calluses of agomiR-6979-5p animals on day 4 and day 7 by qRT-PCR analysis. n=3, per group. Mice treated with agomiR-6979-5p exhibited a smaller callus volume and a disrupted fracture gap relative to control animals upon X ray **(E)** and three dimensional m-CT analyses **(F)**. **(G)** Smaller total and bone callus volume can be seen in agomiR-6979-5p-treated animals relative to control animals on post-fracture days 14 and 21 by micro-CT data analysis. **(H)** H&E/Alcian-blue staining results revealed a reduced bone area and increased cartilage area at the fracture junction in the agomiR-6979-5p group. Data are means ± SD of triplicate experiments. *p < 0.05, **p < 0.01, ***p < 0.001.

To explore how miR-6979-5p affects fracture healing, we measured the relative levels of miR-6979-5p in fracture gene chips **(Figure 1A)**. In addition, we generated a murine fracture model, and calluses from fracture sites were harvested to measure miR-6979-5p levels during early fracture healing stages (days 1 to 21) **(Figure 1B)**, revealing this levels to be markedly decreased during this early healing period. Next, mice were divided randomly into two groups (control group and agomiR-6979-5p group), with agomiR-6979-5p animals receiving local direct injections of Cy3-labeled miR-6979-5p at the fracture sites on day 0, day 4, and day 7 post-injury, after which miR-6979-5p levels in fracture sites were assessed via *in vivo* imaging in these animals **(Figure 1C).** On days 4, 7, 14 and 21 post-injury, 3 mice in each group were euthanatized and calluses were collected from the fracture sites to measure the level of miR-6979-5p and, as shown in **Figure 1D,** miR-6979-5p levels were higher in mice administered agomiR-6979-5p than in control animals at both of these time points. All animals were also assessed via X-rays and micro-CT examinations during the fracture healing process, and those animals treated with agomiR-6979-5p exhibited a smaller callus volume and a larger fracture gap relative to control animals **(Figure 1E-F).** Additionally, micro-CT scanning datas indicated the presence of a smaller total and bone callus volume in agomiR-6979-5p-treated animals than in control animals on post-fracture days 14 and 21 **(Figure 1G).** Moreover, H&E/Alcian-blue staining results revealed a reduced bone area and increased cartilage area at the fracture junction in the agomiR-6979-5p group, suggesting less bone remodeling for these animals **(Figure 1H).** These results indicated that miR-6979-5p may negatively regulate the fracture healing process.

### MiR-6979-5p negatively regulates in vitro osteoblast differentiation

**Figure 2:**
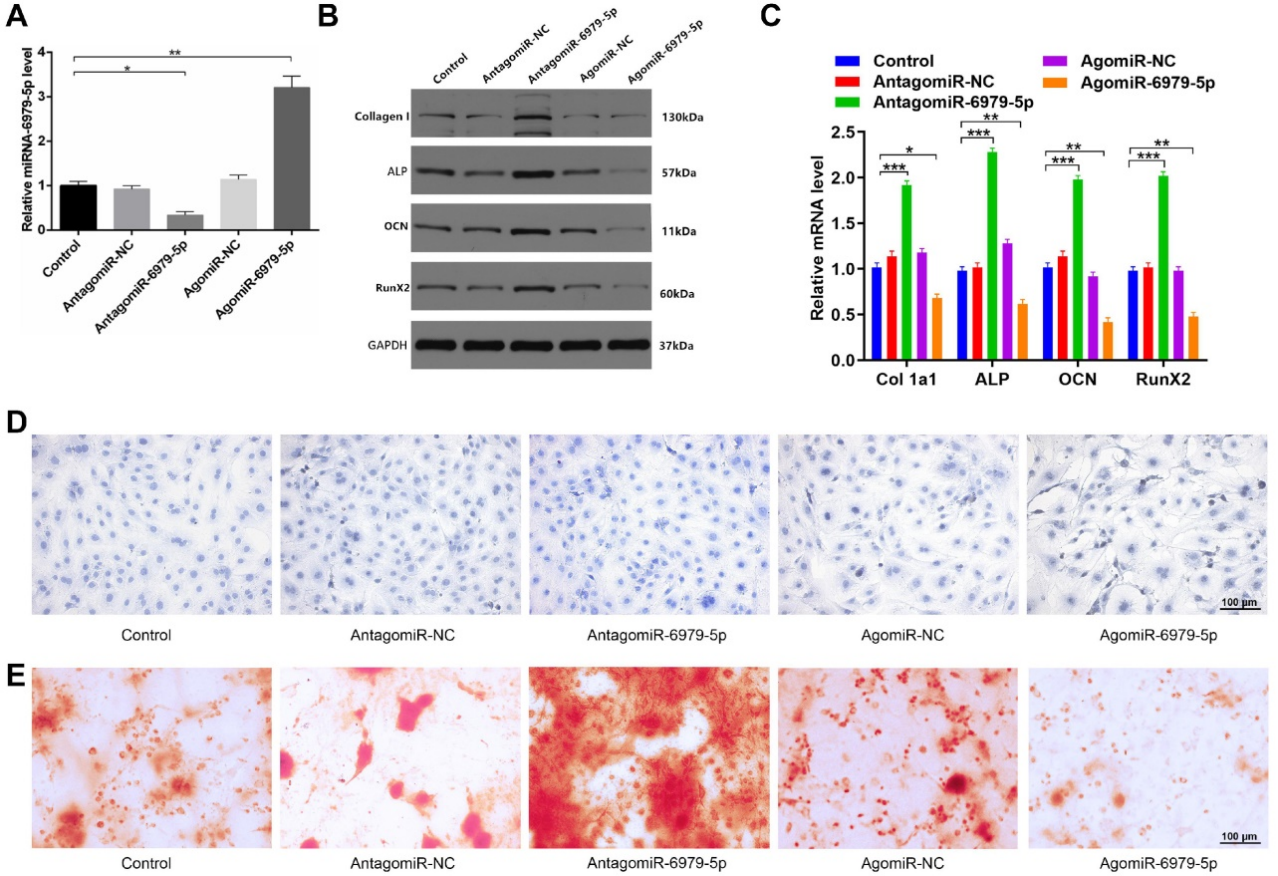
** miR-6979-5p inhibits osteoblast differentiation *in vitro.***MC3T3-E1 cells were transfected with lipofectamine 3000 only, antagomiR-NC, antagomiR-6979-5p, agomiR-NC, or agomiR-6979-5p. MiR-6979-5p levels were up-regulated in cells treated with agomiR-6979-5p as measured via qRT-PCR analysis. **(B-C)** Western blotting and qRT-PCR analyses of the osteogenic markers among the treated groups**.** ALP staining **(D)** and alizarin red-mediated calcium staining in **(E)** for cells treated as in **(A)**. Scale bar, 100 µm. Data are means ± SD of triplicate experiments. *p < 0.05, **p < 0.01, ***p < 0.001.

We next explored how miR-6979-5p affects osteogenic differentiation *in vitro*. MC3T3-E1 cells were transfected with lipofectamine 3000 alone, or together with an antagomiR-negative control (antagomiR-NC), antagomiR-6979-5p, agomiR-negative control (agomiR-NC), or agomiR-6979-5p. Overexpression of miR-6979-5p was evident in the agomiR-6979-5p group by PCR analysis **(Figure 2A)**. Next, expression of collagen I, ALP, OCN, and RunX2, which are osteogenic genes, were measured among the five groups via western blotting. As shown in **Figure 2B and 2C**, higher mRNA levels of relative osteogenic genes were detectable in the antagomiR-6979-5p group than in the other experimental groups. Furthermore, we found overexpression of miR-6979-5p reduced ALP activity and staining, whereas knockdown of miR-6979-5p via antagomiR-6979-5p transfection enhanced ALP activity and staining **(Figure 2D)**. In addition, to assess whether miR-6979-5p stimulates or suppresses extracellular matrix mineralization, cells were continuously cultured for 21 days, after which we observed reduced mineral deposition in agomiR-6979-5p-transfected cells relative to antagomiR-6979-5p-transfected cells **(Figure 2E)**. These data indicated that miR-6979-5p inhibits the osteoblast differentiation of MC3T3-E1 cells.

### MiR-6979-5p regulates osteogenic differentiation via directly targeting BMP2

**Figure 3:**
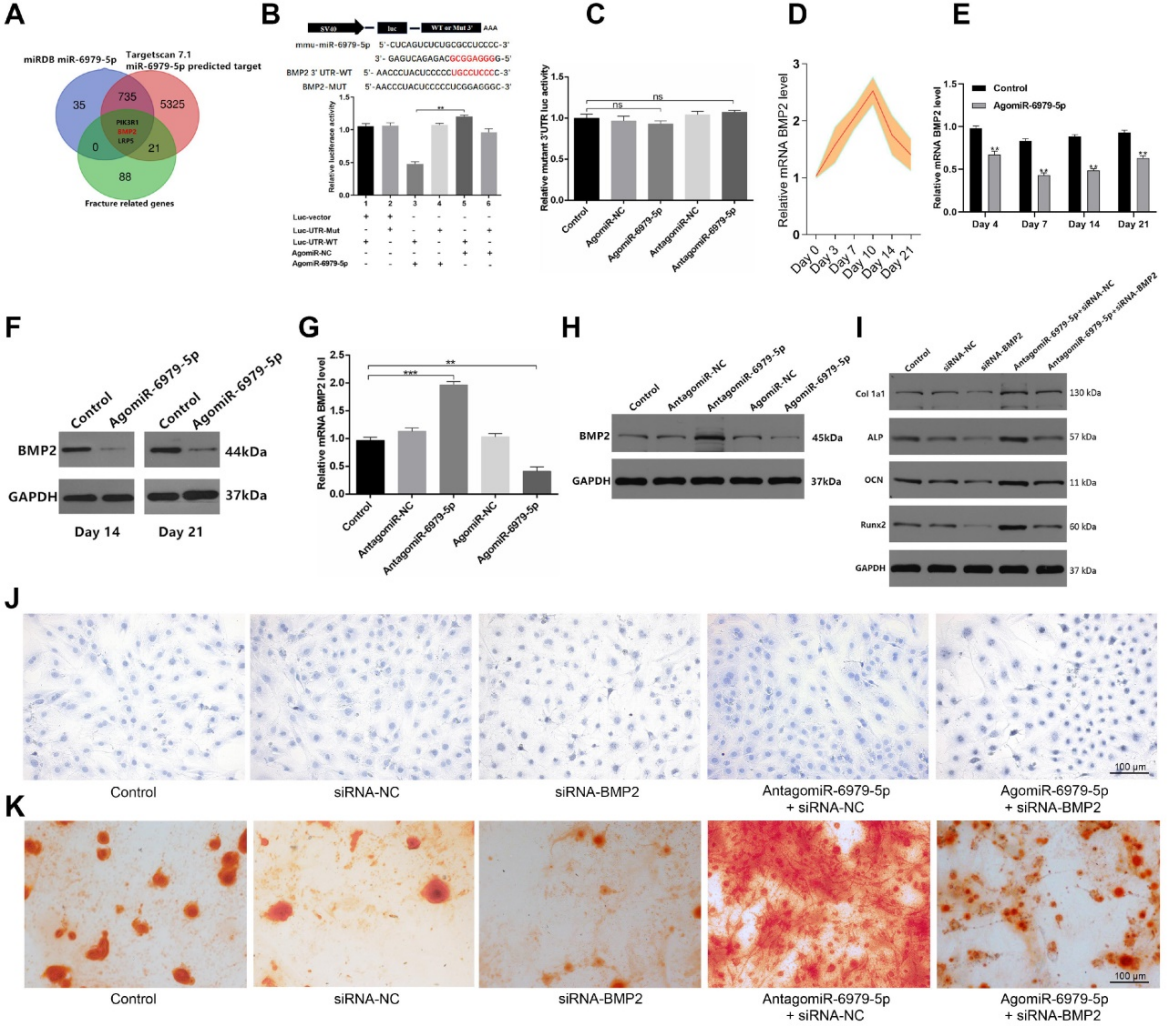
** BMP2 is a miR-6979-5p target gene.** Three genes (PI3KR1, BMP2, and LRP5) were selected as potential targets of miR-6979-5p. **(B)** AgomiR-6979-5p substantially attenuated WT BMP2 3' UTR reporter activity.** (C)** No significant differential activity of the mutated 3' UTR BMP2 reporter was evident between groups. **(D)** Elevated levels of BMP2 in calluses of fracture model animals were detectable at the early stage of fracture healing (days 1 to 21). **(E)** Lower BMP2 mRNA levels in calluses samples were detected by qRT-PCR analysis in agomiR-6979-5p-treated animals than in those in the other groups at time day points (days 4, 7, 14, and 21). **(F)** Western blotting of BMP2 in calluses samples between the two groups on day 14 and day 21. **(G)** qRT**-**PCR analysis of BMP2 mRNA levels in the five transfected groups (Control group, agomiR-NC group, agomiR-6979-5p group, antagomiR-NC group, and antagomiR-6979-5p). **(H)** Western blotting analysis of BMP2 mRNA levels in **(G)**.** (I)** Western blotting of collagen I, ALP, OCN, and Runx2 mRNA levels in five groups (Control group, siRNA-NC, siRNA-BMP2 group, agomiR-6979-5p+siRNA-BMP2, and agomiR-6979-5p+siRNA-NC group). ALP staining **(J)** and alizarin red-mediated calcium staining **(K)** results in** (A)**. Scale bar, 100 µm. Data are means ± SD of triplicate experiments. *p < 0.05, **p < 0.01, ***p < 0.001.

We next sought to explore miR-6979-5p downstream targets via screening miRDB datasets, Targetscan datasets, and GEO datasets for potential targets. we combined the results of the above three databases to enhance the strength of the evidence, and in order to show the process of the method of selecting target gene more clearly, we produced a Venn diagram and as shown in Figure 3A, three candidate genes were identified of interest - PI3KR1, BMP2, and LRP5. Following a literature review, although LRP5 is involved in Wnt/LRP5/β-Catenin signaling pathway, and thereby mediate the osteoblast differentiation process 22, BMP2 is a osteogenic mediator demonstrated widely in much more previous researches 23-25. Thus, we select BMP2 from the three candidates as a target gene for miR-6979-5p. We next assessed whether miR-6979-5p directly targets BMP2, using either wild-type (WT) BMP2 3′ UTR or mutated (Mut) BMP2 3' UTR constructs fused to luciferase reporters. With these constructs, we found that agomiR-6979-5p substantially attenuated WT BMP2 3′ UTR reporter activity **(Figure 3B)**, whereas it failed to influence the activity of the mutated 3′ UTR BMP2 reporter **(Figure 3C)**. Furthermore, BMP2 mRNA levels in fracture calluses were measured over a 21 day post-injury period, revealing an apparent increase in expression over time **(Figure 3D).** To assess changes in BMP2 mRNA levels during the early stages of fracture healing *in vivo*, calluses samples from both animal groups were collected on days 4, 7, 14, and 21 for PCR analysis. Lower BMP2 expression was evidence in agomiR-6979-5p-treated animals relative to control animals at each time point **(Figure 3E)**. Similarly, fracture calluses were collected from both animal groups on days 14 and 21 for western blotting, and the results indicated that BMP2 expression was significant lower in the agomiR-6979-5p group relative to controls **(Figure 3F)**. Next, MC3T3-E1 cells were transfected using either agomiR or antagomiR constructs as above, and BMP2 mRNA expression was assessed via PCR and western blotting. As shown in **Figure 3G-H**, higher BMP2 mRNA levels were evident in the antagomiR-6979-5p group relative to other experimental groups. Additionally, to explore whether osteoblast differentiation is BMP2-dependent, we next tested whether antagomiR-6979-5p was able to rescue the negative effects of siRNA-BMP2 on osteogenic differentiation. Western blotting indicated that antagomiR-6979-5p was able to restore BMP2-dependent osteogenesis-associated gene expression (collagen I, ALP, OCN, and Runx2) **(Figure 3I)**. Moreover, we found overexpression of BMP induced ALP activity and staining, whereas antagomiR-6979-5p was able to restore the effect of BMP2 on ALP activity and staining **(Figure 3J)**. To observe whether miR-6979-5p stimulates or suppresses extracellular matrix mineralization, cells were next continuously cultured for 21 days, and we observed reduced mineral deposition in siBMP2-transfected cells relative to the antagomiR-6979-5p+siRNA-NC group **(Figure 3K)**. Collectively, these results indicated miR-6979-5p directly targets the BMP2 3' UTR in the context of osteogenic differentiation.

### LncRNA Rhno1 serves to sequester miR-6979-5p

**Figure 4:**
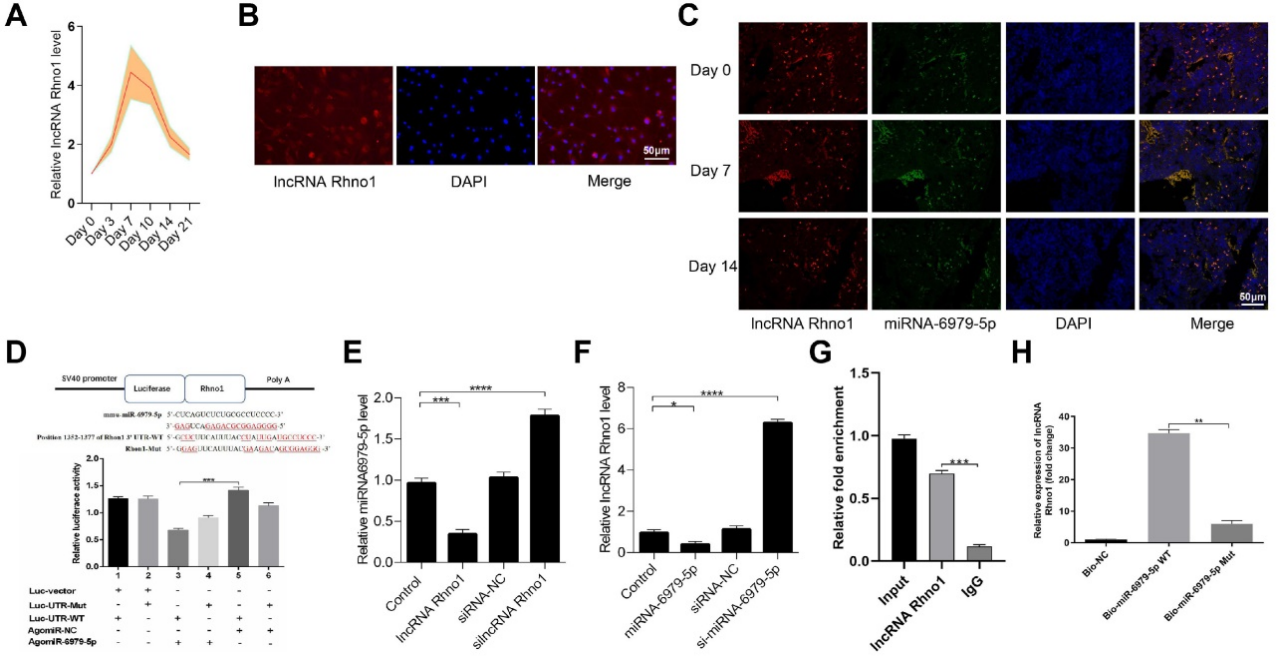
** LncRNA Rhno1 suppressed miR-6979-5p expression by sequestering this miRNA MC3T3-E1 cells.** Levels of Rhno1 are increased in fracture animal models at early stages of fracture healing. **(B)** Localization of lncRNA Rhno1 by RNA-FISH in MC3T3-E1 cells. LncRNA Rhno1 is stained red (DAPI), and nuclei are stained blue (Scale bar = 50 µm). **(C)** Localization of lncRNA Rhno1 by RNA-FISH in bone tissues and calluses on days 7 and 14 post injury. LncRNA Rhno1 is stained red (DAPI), miR-6979-5p is stained green (DAPI) and nuclei are stained blue (Scale bar = 50 µm).** (D)** AgomiR-6979-5p substantially attenuated WT lncRNA Rhno1 3' UTR reporter activity. **(E)** miR-6979-5p levels in silncRNA Rhno1-treated samples were significantly higher than other groups. **(F)** Similarly, lncRNA Rhno1 levels were much higher in the antagomiR-6979-5p-treated group than in other groups, particularly relative to the agomiR-6979-5p group. **(G)** The interaction of miR-6979-5p and lncRNA Rhno1 with Ago2 was assessed via RIP assays. RNA levels were presented as fold enrichment in Ago2 relative to IgG immunoprecipitates (lower panel). **(H)** qRT-PCR was used to detect lncRNA Rhno1 expression in the sample pulled down by biotinylated miR-6979-5p WT and miR-6979-5p Mut probes. Data are means ± SD of triplicate experiments. *p < 0.05, **p < 0.01, ***p < 0.001.

Next, to further elucidate the upstream regulatory mechanisms of miR-6979-5p, we searched the LncBase Experimental V.2 Database to identify its putative regulators, and a possible interaction between the lncRNA Rhno1 and miR-6979-5p was found. Consistent with this, in fracture animal models we found the level of lncRNA Rhno1 to trend upwards during the early stages of fracture healing (day 1 to 21 post-injury) **(Figure 4A).** Next, we assessed lncRNA Rhno1 localization in MC3T3-E1 cells via fluorescence in situ hybridization (FISH), revealing that lncRNA Rhno1 is localized mainly in the cytoplasm of MC3T3-E1 cells **(Figure 4B).** Furthermore, FISH assays were used to assess lncRNA Rhno1 localization in bone or calluses among animal models on days 0, 7, 14 post-injury, which suggesting lncRNA Rhno1 is localized mainly in the cytoplasm of bone tissues or calluses **(Figure 4C).** Moreover, using StarBase v2.0 (http://starbase.sysu.edu.cn/starbase2/mirLncRNA.php), we were able to identify the site of interaction between lncRNA Rhno1 and miR-6979-5p as shown in **Figure 4D.** A luciferase reporter gene assay was next conducted, and results in **Figure 4D** confirmed that the lncRNA Rhno1 directly bound to miR-6979-5p in MC3T3-E1 cells. To further validate the association between lncRNA Rhno1 and miR-6979-5p, MC3T3-E1 cells were next transfected with lipofectamine 3000 either alone, or together with silncRNA control (silncRNA-NC), or silncRNA Rhno1, and after a 48 hours cell culture period miR-6979-5p levels were measured by qRT-PCR analysis. This led miR-6979-5p levels in the silncRNA Rhno1-treated cells to be significantly higher than in the other experimental groups **(Figure 4E)**. Similarly, the lncRNA Rhno1 levels were much higher in the antagomiR-6979-5p group than other groups, especially relative to the agomiR-6979-5p group **(Figure 4F)**. Furthermore, the interaction between lncRNA Rhno1 and miR-6979-5p was further demonstrated via an RNA RIP assay, which revealed that lncRNA Rhno1 increased Ago2-containing miRNA ribonucleoprotein complex enrichment than when mixed with samples imunoprecipitated using control IgG **(Figure 4G)**. Consistent with this, a pull-down assay provided evidence that lncRNA Rhno1 was readily bound by biomiR-6979-5p **(Figure 4H)**, but not by a mutated isoform of this bio-miR-6979-5p. Collectively, these results suggest that lncRNA Rhno1 may serve as a ceRNA that functions as a molecular sponge to sequester miR-6979-5p.

### LncRNA Rhno1 promotes *in vitro* osteoblast differentiation

**Figure 5:**
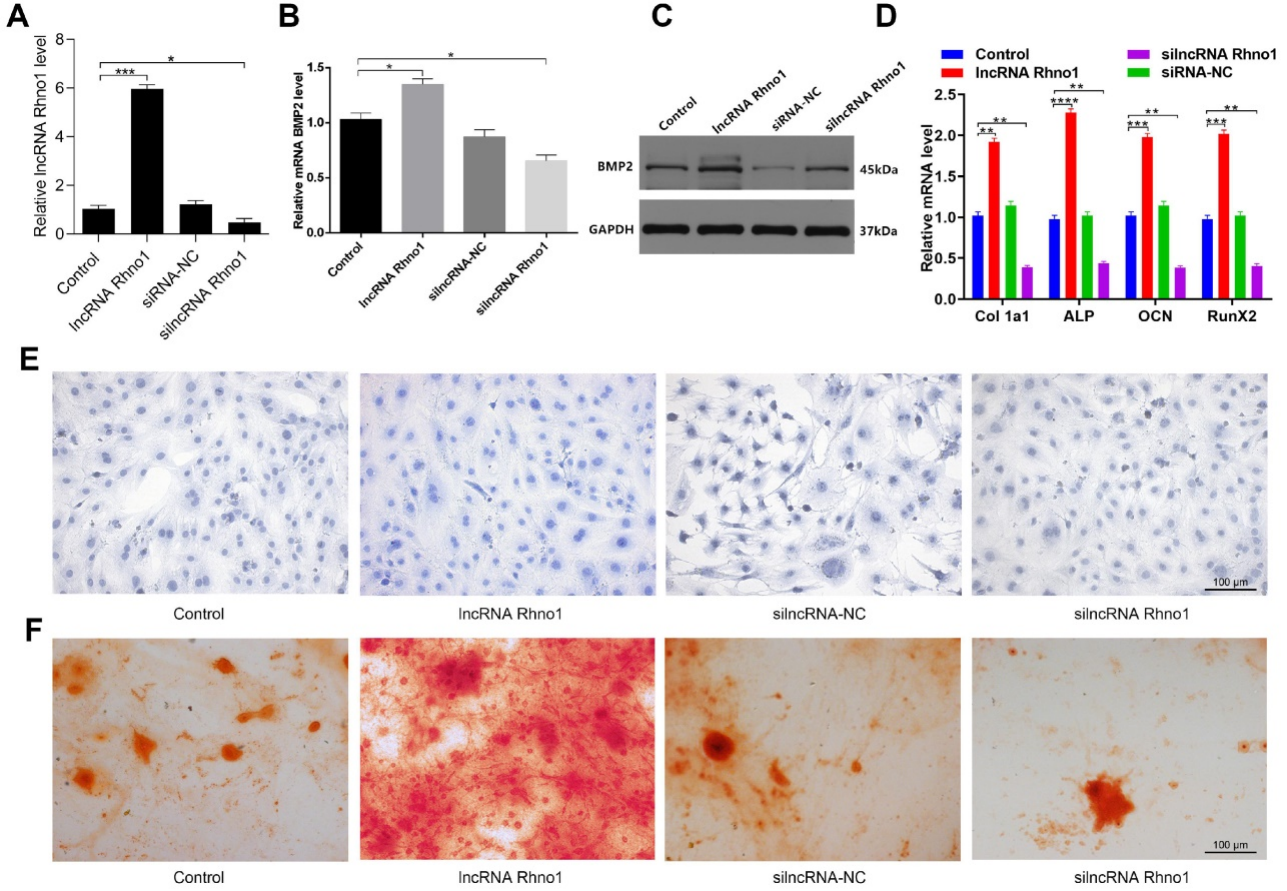
** LncRNA Rhno1 promotes the osteoblastic differentiation of MC3T3-E1 cells.** MC3T3-E1 cells were transfected with lipofectamine 3000 only, lncRNA Rhno1, siRNA-NC, or silncRNA Rhno1. Overexpression of lncRNA Rhno1 was detectable in lncRNA Rhno1-transfected cells via qRT-PCR analysis. **(B-C)** Overexpression of BMP2 were detectable in lncRNA Rhno1-transfected cells in **(A)**. **(D)** Collagen I, ALP, OCN, and RunX2 were upregulated in** (A).** ALP staining **(E)** and alizarin red-mediated calcium staining **(F)** in MC3T3-E1 cells treated as in **(A)**. Scale bar, 100 µm. Data are means ± SD of triplicate experiments. *p < 0.05, **p < 0.01, ***p < 0.001.

To explore how the lncRNA Rhno1 influences osteogenic differentiation, MC3T3-E1 cells were treated with lipofectamine 3000 alone, or together with lncRNA Rhno1, silncRNA-NC, or silncRNA Rhno1 **(Figure 5A).** After continuous culture for 48 hours, overexpression of lncRNA Rhno1 and BMP2 was then detectable in lncRNA Rhno1-transfected cells by qRT-PCR analysis **(Figure 5B).** The results of western blotting analyses of BMP2 levels in these four groups were also consistent with the qRT-PCR results **(Figure 5C).** Consistent with these changes, we found that the expression of genes involved in osteoblast differentiation, such as collagen I, ALP, OCN, and RunX2, were elevated in the lncRNA Rhno1 group relative to the other groups **(Figure 5D, Supplementary Figure 1)**. In addition, overexpression of lncRNA Rhno1 induced ALP activity and staining, whereas lncRNA Rhno1 silencing attenuated ALP activity and staining **(Figure 5E)**. Moreover, to observe the effect of lncRNA Rhno1 on extracellular matrix mineralization, cells were continuously cultured for 21 days, after which we observed more mineral deposition in lncRNA Rhno1-transfected cells **(Figure 5F)**. This indicates that lncRNA Rhno1 enhances the expression of BMP2 and exerts a positive role during osteogenic differentiation.

### LncRNA Rhno1 accelerates fracture healing *in vivo*

**Figure 6:**
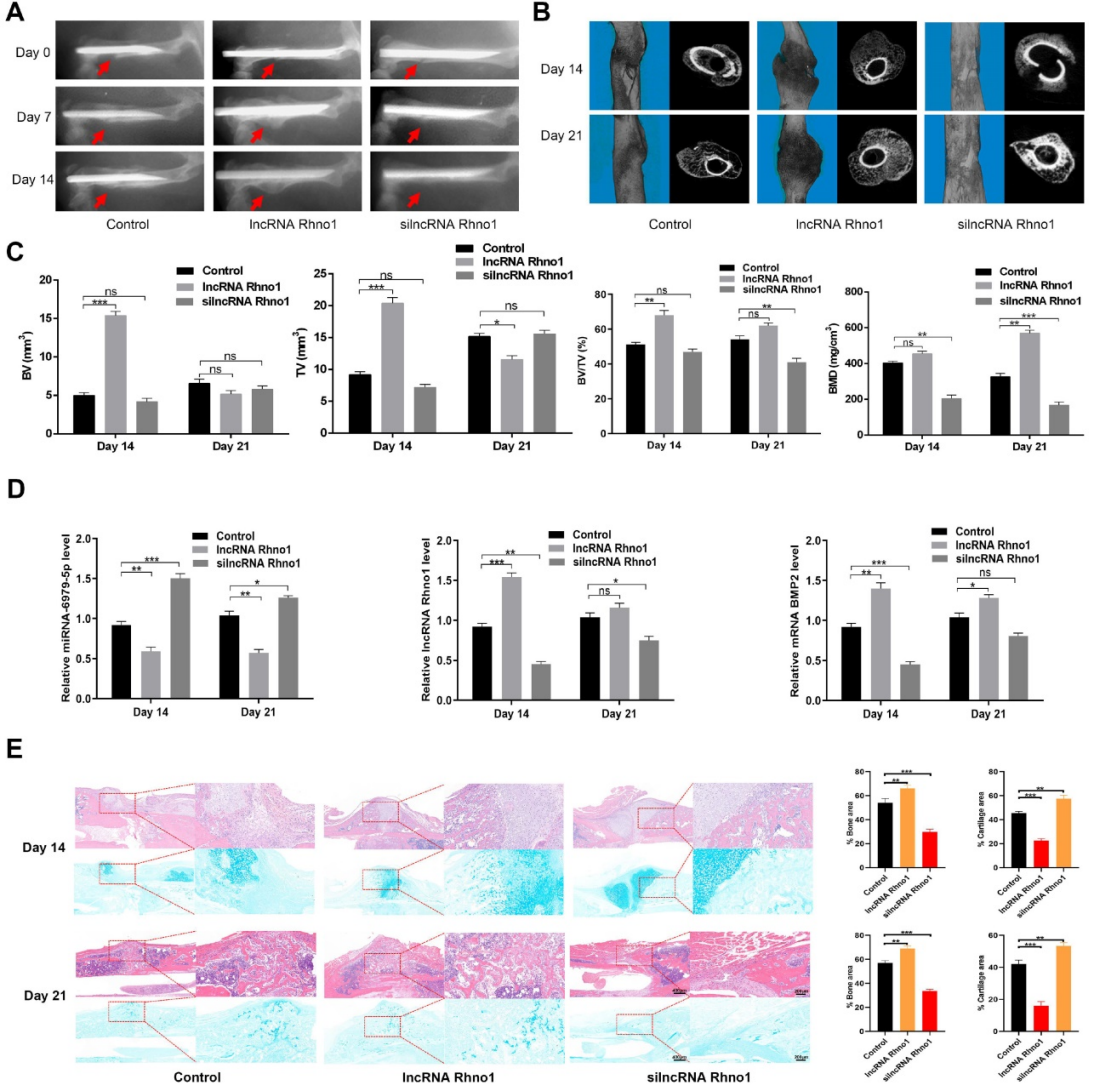
** Local administration of lncRNA Rhno1 promotes fracture healing in mice.** X ray comparisons of fracture healing between control, lncRNA Rhno1, and silncRNA Rhno1 treated animals at days 7, 14, and 21 post-operation. **(B)** micro-CT images in **(A)** at day 14, and 21 after injury. **(C)** BV, TV, and BV/TV of the callus in **(A)** on days 14 and 21 post-operation were established via micro-CT. n=5 mice, per group. **(D)** The levels of miR-6979-5p were decreased in lncRNA Rhno1-treated animals, whereas the levels of BMP2 and lncRNA Rhno1 increased in these samples, as measured by qRT-PCR analysis. **(E)** H&E/Alcian-blue staining results revealed a reduced cartilage area and increased bone area at the fracture junction in the lncRNA Rhno1 group. Data are means ± SD of triplicate experiments. *p < 0.05, **p < 0.01, ***p < 0.001.

To evaluate the effects of lncRNA Rhno1 on fracture healing, fracture model mice were locally injected with phosphate buffered saline (PBS), lncRNA Rhno1, or an equal volume of silncRNA Rhno1 in the fracture sites on days 0, 4, and 7 after surgery. X rays and CT examinations were performed to assess the process of fracture healing. Mice treated with lncRNA Rhno1 exhibited a larger callus volume and smaller fracture gap relative to control animals **(Figure 6A-B)**. In addition, a larger total and bone callus volume was evident in lncRNA Rhno1-treated animals relative to other groups on post-fracture days 14 and 21, whereas silncRNA Rhno1-treated animals had the smallest total and bone callus volume among the three groups **(Figure 6C)**. Furthermore, on days 14 and 21 post micro-CT scanning, 3 mice from each group were euthanatized and calluses were collected to measure the levels of miR-6979-5p, BMP2, and lncRNA Rhno1, and as shown in **Figure 6D**, the level of miR-6979-5p decreased in lncRNA Rhno1-treated animals, whereas the levels of BMP2 and lncRNA Rhno1 increased. Moreover, H&E/Alcian-blue staining results revealed a reduced cartilage area and increased bone area at the fracture junction in the lncRNA Rhno1 group, suggesting more bone remodeling for these animals **(Figure 6E)**. In summary, these data suggest that lncRNA Rhno1 downregulates miR-6979-5p and induces the expression of BMP2, thereby promoting fracture healing *in vivo*.

## Discussion

**Figure 7:**
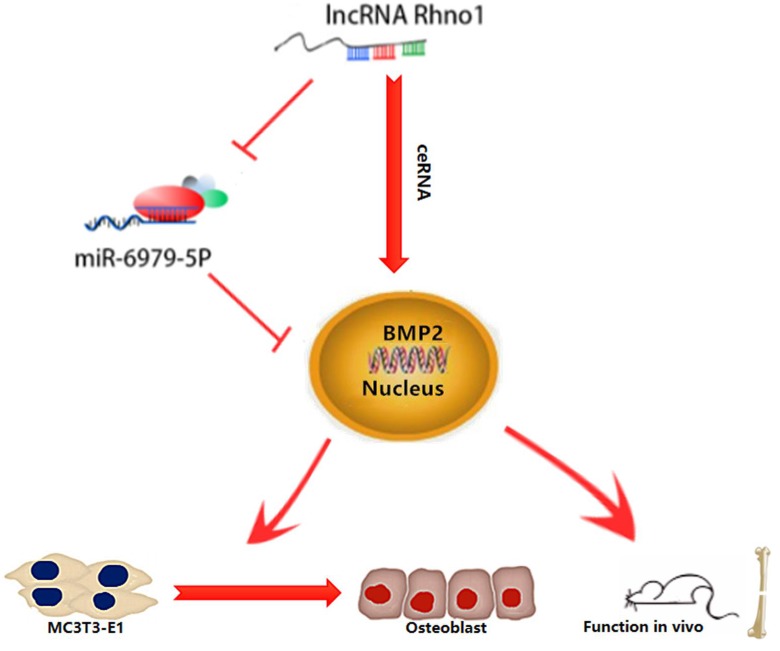
** A model of the lncRNA Rhno1/miR-6979-5p/BMP2 axis regulation of osteoblast differentiation.** LncRNA Rhno1 serves as a ceRNA that competitively inhibits the expression of miR-6979-5p, thereby elevating the BMP2 mRNA level, and thus promoting osteoblast differentiation.

This study provides the first verification that local injection of lncRNA Rhno1 into murine fracture sites induces a prominent regenerative effect on the bone and its micro-architecture, as evidenced by more rapid healing and larger callus volumes in treated animals. We further found *in vitro* that lncRNA Rhno1 could serve as a ceRNA for miR-6979-5p, thereby counteracting the negative effects of miR-6979-5p on osteoblast differentiation and fracture healing. Moreover, we explored miR-6979-5p downstream targets, revealing BMP2 to be a relevant target gene for this miRNA. Thus, as shown in **Figure 7**, our results suggest that lncRNA Rhno1 may induce osteoblast differentiation by competitively binding miR-6979-5p to upregulate BMP2 mRNA levels, and thus lncRNA Rhno1/miR-6979-5p/BMP2 axis plays a significant role in the regulation of osteoblast differentiation.

In recent decades, miRNAs have been reported to serve as important regulatory factors in the context of osteogenic differentiation, and the underlying mechanisms have been the focus of substantial research 26-29. We therefore sought to identify miRNAs with differential expression between intact control bone and calluses of post-fracture mice, leading to our selection of miR-6979-5p from the GEO database. We found no articles regarding the function of miR-6979-5p in the reported literature, and thus it was necessary to investigate the biological functions of this miRNA. In this study, the effect of miR-6979-5p on fracture healing was investigated *in vivo*, with the results suggesting that miR-6979-5p inhibits fracture healing, and we were able to confirm this negative impact on osteogenic differentiation using MC3T3-E1 cells. As previous studies 30 have reported miRNAs can bind to target mRNAs to exert specific effects, we subsequently explored the downstream targets of miR-6979-5p using luciferase assays.

BMP2, which we confirmed to be a target for miR-6979-5p, is able to stimulate the differentiation of myoblasts into osteoblasts via the EIF2AK3-EIF2A-ATF4 pathway 31. The activation of EIF2AK3 can in turn stimulate phosphorylation of EIF2A, leading to increased ATF4 expression, which plays a central role in osteoblast differentiation 32,33. Indeed, several studies have previously demonstrated that BMP2 plays a regulatory role in various biological processes 34-36. For example, one group found that a BMP activity gradient is essential for normal dorsoventral patterning during gastrulation 37. In our study, we demonstrated that BMP2 is a direct target of miR-6979-5p, and downregulation of miR-6979-5p increases the BMP2 mRNA levels, thereby promoting osteogenesis.

In addition, lncRNAs are known to affect disease progression by serving as molecular ceRNA sponges that can sequester miRNAs 38. For example, one group found that the lncRNA AGAP2-AS1 plays a competitive role in hepatocellular carcinoma via acting as a miR-16-5p sponge 39. In present study, we searched for potential upstream regulators of miR-6979-5p using the LncBase Experimental V.2 Database (http://carolina.imis.athena-innovation.gr/diana_tools/web/index.php?r=lncbasev2%2Findex-experimental), leading to our identification of the lncRNA Rhno1. As a growing body of evidence suggests that cytoplasmic lncRNAs can serve as decoys for other noncoding RNAs such as miRNAs 40,41, we determined the localization of this lncRNA Rhno1 via a FISH approach, revealing that lncRNA Rhno1 mainly located in cytoplasm, which is consistent with other published findings. RIP and pull down assays further confirmed the ability of lncRNA Rhno1 and miR-6979-5p to directly interact, thereby providing additional support to our model wherein the lncRNA Rhno1/miR-6979-5p/BMP2 axis modulates osteoblast differentiation.

There were a number of limitations in the present study. First, it remains to be determined whether more frequent injections of lncRNA Rhno1 can induce similar or much better bone formation effects in fracture mice. Moreover, we were not able to conduct clinical trials regarding the beneficial effects of lncRNA Rhno1 on fracture healing in the present study. In addition, to produce more definitive evidence of the role of the lncRNA Rhno1/miR-6979-5p/BMP2 axis in osteogenesis, transgenic mice that specifically lack relevant genes in osteoblast lineages are necessary.

Taken together, our results indicate that the lncRNA Rhno1/miR-6979-5p/BMP2 axis can effectively modulate the differentiation of osteoblasts; potentially due to the lncRNA Rhno1 serving as a ceRNA that competitively inhibits the expression of miR-6979-5p, thereby elevating BMP2 mRNA levels and promoting osteoblast differentiation. Our findings suggest that the lncRNA Rhno1/miR-6979-5p/BMP2 axis may represent a novel therapeutic target for accelerating fracture healing.

## Supplementary Material

Supplementary figure S1.

## Figures and Tables

**Table 1:** miRNAs, lncRNAs, and mRNA primer sequence

microRNAs or gene name	Primer sequence
Mmu - miR - 6979 - 5p - Forward	ACACTCCAGCTGGGGGGGAGGCGCAGAG
Mmu - miR - 6979 - 5p - Reverse	TGGTGTCGTGGAGTCG
Mmu - lncRNA - Rhno1 - Forward	CTATGAGACAGGAGGCAGGGT
Mmu - lncRNA - Rhno1 - Reverse	AATGTGAGGCCCTTTTGTCTCA
U6-Forward	CTCGCTTCGGCAGCACA
U6-Reverse	AACGCTTCACGAATTTGCGT
Mmu - BMP2 - Forward	GACATCCTGAGCGAGTTCGA
Mmu - BMP2 - Reverse	CACTTGTTTCTGGCAGTTCTTC
Mmu - ALP - Forward	TGACTACCACTCGGGTGAACC
Mmu - ALP - Reverse	TGATATGCGATGTCCTTGCAG
Mmu - COL1A1 - Forward	CTGACTGGAAGAGCGGAGAG
Mmu - COL1A1 - Reverse	CGGCTGAGTAGGGAACACAC
Mmu - OCN - Forward	TTCTGCTCACTCTGCTGACCC
Mmu - OCN - Reverse	CTGATAGCTCGTCACAAGCAGG
Mmu - Runx2 - Forward	CGCCACCACTCACTACCACAC
Mmu - Runx2 - Reverse	TGGATTTAATAGCGTGCTGCC
Mmu - GAPDH - Forward	AGAGTGTTTCCTCGTCCCG
Mmu - GAPDH - Reverse	CCGTTGAATTTGCCGTGA
