# Supplementary material for: The lncRNA Rhno1/miR-6979-5p/BMP2 Axis Modulates Osteoblast Differentiation
Source: Int J Biol Sci. 2020 Mar 12;16(9):1604–15. doi: 10.7150/ijbs.38930 (PMC7097916; doi:10.7150/ijbs.38930)
Supplement: Supplementary file 1 — Supplementary figure S1. [file ijbsv16p1604s1.pdf]

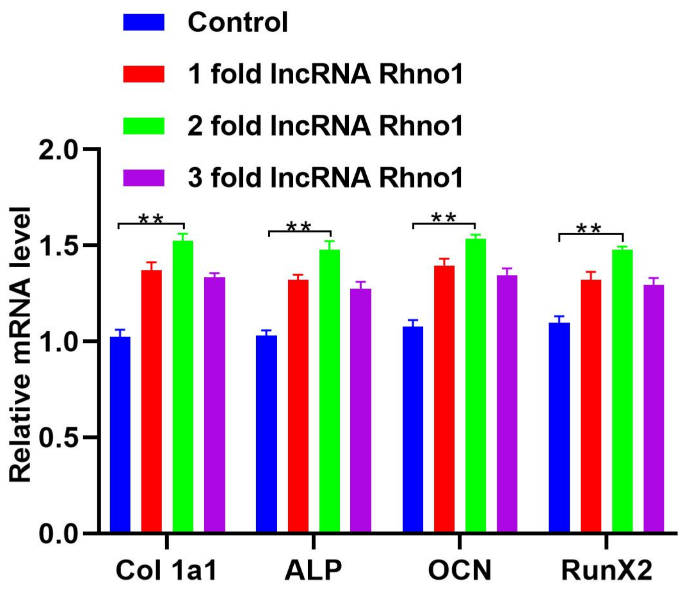

Figure S1. qRT-PCR results of the expression of osteogenic genes in MC3T3-E1 cells with different concentration of lncRNA Rhno1 treatment.
